# Prognostic value of plasma Epstein–Barr virus DNA level during posttreatment follow-up in the patients with nasopharyngeal carcinoma having undergone intensity-modulated radiotherapy

**DOI:** 10.1186/s40880-017-0256-x

**Published:** 2017-11-07

**Authors:** Wen-Fei Li, Yuan Zhang, Xiao-Bin Huang, Xiao-Jing Du, Ling-Long Tang, Lei Chen, Hao Peng, Rui Guo, Ying Sun, Jun Ma

**Affiliations:** 1 Department of Radiation Oncology, Sun Yat-sen University Cancer Center; State Key Laboratory of Oncology in South China; Collaborative Innovation Center for Cancer Medicine, 651 Dongfeng Road East, Guangzhou, 510060 Guangdong P. R. China; 2 Department of Blood Source Management, Guangzhou Blood Center, Guangzhou, 510095 Guangdong P. R. China

**Keywords:** Nasopharyngeal carcinoma, Epstein–Barr virus DNA, Follow-up, Tumor recurrence

## Abstract

**Background:**

The value of Epstein–Barr virus (EBV) DNA assay during posttreatment follow-up of the patients with nasopharyngeal carcinoma (NPC) presenting with different pretreatment plasma EBV DNA levels remains unclear. In the present study, we aimed to evaluate the prognostic value of plasma EBV DNA assay during posttreatment follow-up in the patients with NPC who have undergone intensity-modulated radiotherapy.

**Methods:**

The medical records of 385 NPC patients treated with intensity-modulated radiotherapy between November 2009 and February 2012 were reviewed. All patients underwent plasma EBV DNA assays before treatment, within 3 months after treatment, and then every 3–12 months during posttreatment follow-up period. The recurrence rates for patients with different pretreatment and posttreatment follow-up plasma EBV DNA levels were analyzed.

**Results:**

Of the 385 patients, 267 (69.4%) had detectable pretreatment plasma EBV DNA (> 0 copy/mL) and 93 (24.2%) had detectable posttreatment EBV DNA during a median follow-up of 52.8 months (range 9.3–73.8 months). Detectable EBV DNA during posttreatment follow-up was found in 14.4% (17/118) and 28.5% (76/267) of patients with undetectable and detectable pretreatment EBV DNA, respectively, and was significantly associated with tumor recurrence in both patient groups. EBV DNA was detectable in 12.8% (40/313) of patients who remained disease-free, 56.4% (22/39) of patients with locoregional recurrence alone, and 93.9% (31/33) of patients with distant metastasis as the first recurrence event (*P* < 0.001); 6.5% (19/292) of patients with undetectable EBV DNA and 57.0% (53/93) of patient with detectable EBV DNA during posttreatment follow-up experienced tumor recurrence. Compared with other cut-off values, the cut-off value of 0 copy/mL for EBV DNA during posttreatment follow-up had the highest area under the ROC curve (AUC) value (0.804, 95% confidence interval 0.741–0.868) for predicting tumor recurrence (sensitivity, specificity, and accuracy: 73.6%, 87.2%, and 84.7%, respectively).

**Conclusion:**

Plasma EBV DNA level during posttreatment follow-up is a good marker for predicting distant metastasis but not locoregional recurrence in the patients with NPC irrespective of the pretreatment EBV DNA levels.

## Background

Nasopharyngeal carcinoma (NPC) is a common head and neck cancer in China, with 60,600 new cases reported in 2015 [1]. The incidence varies in different areas of China; the highest risk areas are South China, especially Guangdong Province; low rates of NPC are generally observed in North China [2]. Epstein–Barr virus (EBV) infection is an important etiological factor in NPC. Recent studies indicated that circulating EBV DNA originates from tumor lesions and associates with tumor load, and the plasma EBV DNA assay is widely used for screening, prognostic prediction, and post-treatment surveillance of patients with NPC [3, 4]. Pretreatment plasma EBV DNA was detectable (> 0 copy/mL) in 69%–97% of patients with NPC and was closely related to clinical stage and treatment outcome [5–17]. On the other hand, plasma EBV DNA detected within 3 months after treatment was found in 4.3%–30% of patients with NPC and was associated with short survival [6, 12–22].

The plasma EBV DNA assay has also be used to monitor tumor recurrence during posttreatment follow-up of NPC patients. It has been reported that patients with NPC who remained in remission after radiotherapy had consistently undetectable or extremely low levels of plasma EBV DNA, whereas patients who developed recurrence exhibited significantly elevated plasma EBV DNA levels [4, 9, 14, 23–30]. If plasma EBV DNA levels remain elevated after treatment or initially dropped but subsequently increased, the patients are suggested to take a positron emission tomography/computed tomography (CT) (PET/CT) scan to locate the potential site of recurrence [27]. Moreover, it has been reported that in patients with undetectable EBV DNA levels during posttreatment follow-up period but with radiographic evidence of disease recurrence, recurrence was not detected using histologic examination [27, 28]. Therefore, some investigators indicated that applying an EBV DNA screening followed by a PET/CT scan may be a cost-effective follow-up examination for patients with NPC [27].

However, in the clinic, some patients with consistently undetectable plasma EBV DNA during posttreatment follow-up period also develop tumor recurrence, whereas some patients with elevated EBV DNA levels remain disease-free even after long-term follow-up [29]. Thus, the recurrence rates for patients with undetectable or detectable plasma EBV DNA during posttreatment follow-up period remain unclear. Moreover, the value of plasma EBV DNA follow-up in patients with undetectable or detectable pretreatment EBV DNA has not yet been assessed. Therefore, we performed this retrospective study to further investigate the value of plasma EBV DNA for predicting tumor recurrence in patients with NPC.

## Patients and methods

### Patients

Medical records of 1811 patients with newly-diagnosed, non-distant metastatic, histologically proven NPC treated with radical intensity-modulated radiotherapy (IMRT) at Sun Yat-sen University Cancer Center (Guangzhou, China) between November 2009 and February 2012 were retrospectively analyzed. All patients underwent physical examination, endoscopy, and conventional imaging scans before treatment, and were restaged according to the 7th edition of the Union for International Cancer Control and American Joint Committee on Cancer (UICC/AJCC) staging system [31]. During the study, institutional guidelines recommended only IMRT for stage I NPC and concurrent chemoradiotherapy with or without neoadjuvant and/or adjuvant chemotherapy for stage II to IVB diseases [17]. Only patients who underwent plasma EBV DNA assays before treatment, within 3 months after treatment, and then every 3–12 months during posttreatment follow-up period were included in this study, and all records of the plasma EBV DNA levels were collected. The authenticity of this article has been validated by uploading the key raw data onto the research data deposit (RDD) public platform (http://www.researchdata.org.cn), with the approval RDD number as RDDA2017000230.

### Quantification of plasma EBV DNA

Samples of peripheral blood (5 mL each) were collected and centrifuged at 1600×*g* for isolation of plasma. DNA from plasma samples was extracted with the QIAamp Blood Kit (Qiagen, Hilden, Germany). In total, 500–1000 µL of each plasma sample were used for DNA extraction per column, with a final elution volume of 50 µL from the extraction column [8]. Plasma EBV DNA levels were measured using a real-time quantitative polymerase chain reaction (qPCR) assay amplifying the *Bam*H I-W region of the EBV genome, as previously described [5, 8]. The sequences of the forward and reverse primers were 5′-GCCAG AGGTA AGTGG ACTTT-3′ and 5′-TACCA CCTCC TCTTC TTGCT-3′, respectively. A dual fluorescence-labelled oligomer 5′-(fluorescein amidite, FAM) CACAC CCAGG CACAC ACTAC ACAT (tetramethylrhodamine, TAMRA)-3′ served as a probe. The plasma EBV DNA level was calculated using the following formula: *C* = *Q* × (*V*_DNA_/*V*_PCR_) × (1/*V*_EXT_), in which *C* represents the target level in plasma (copies/mL), *Q* represents the target quantity (copy number) determined by PCR, *V*_DNA_ represents the total volume of DNA obtained after extraction (typically 50 µL per Qiagen extraction), *V*_PCR_ represents the volume of DNA solution used for PCR (typically 2 µL), and *V*_EXT_ represents the volume of plasma extracted (typically 0.5 mL). A plasma EBV DNA level of higher than 0 copy/mL was considered detectable, whereas a level of 0 copy/mL was considered undetectable.

### Follow-up and assessments

Patients were recommended to undergo examinations at least every 3 months during the first 2 years after IMRT and every 6 months thereafter (or until death). The routine follow-up workup included physical examination, plasma EBV DNA assay, nasopharyngeal fiberoptic endoscopy, nasopharyngeal and neck magnetic resonance imaging (MRI), chest X-ray or CT, liver ultrasound or CT, and whole-body bone scan. If possible, tumor recurrence (locoregional recurrence or distant metastasis) was confirmed by fine needle aspiration or biopsy. For recurrences at sites not accessible, clinical diagnosis was accepted if classical changes were observed using at least two imaging methods with or without clinical symptoms, including ^18^F-fluorodeoxyglucose (^18^F-FDG) PET/CT, MRI, CT, abdominal sonography and/or a whole-body bone scan. To increase diagnostic accuracy in the present study, the diagnoses of tumor recurrence were retrospectively confirmed by two experienced doctors on the basis of abnormal imaging findings and the response to treatment.

### Statistical analysis

Overall survival (OS) was calculated from the end of radiotherapy to death from any cause or censored at last follow-up. Disease-free survival (DFS) was calculated from the end of radiotherapy to the date of locoregional recurrence or distant metastasis, whichever occurred first. Distant metastasis-free survival (DMFS) and locoregional recurrence-free survival (LRRFS) were calculated from the end of radiotherapy to the date of distant metastasis or first locoregional recurrence, respectively. EBV failure-free survival was calculated from the end of radiotherapy to the date of first emergence of plasma EBV DNA during follow-up, which was defined as the first emergence of EBV DNA after radiotherapy to the date of first tumor recurrence or the last follow-up in patients with undetectable or persistently detectable EBV DNA after radiotherapy, or the re-emergence of EBV DNA in patients with transiently detectable EBV DNA after radiotherapy followed by a rapid regression to undetectable levels. Living patients without an event corresponding to any endpoint were censored at the date of last follow-up.

All statistical analyses were performed using SPSS v13.0 (SPSS Inc., Chicago, IL, USA). The Chi square test was used to compare categorical variables (or Fisher’s exact test, if the expected number was < 5 in at least 1 cell) and test the association between EBV DNA levels and recurrence. Receiver operating characteristic (ROC) curves were generated, and the area under the ROC curve (AUC) and 95% confidence interval (CI) was calculated to determine the optimal cut-off value of plasma EBV DNA for predicting tumor recurrence with the best trade-off between sensitivity and specificity. Survival rates were calculated using the Kaplan–Meier method, and the differences were compared using the log-rank test. Two-tailed *P* values < 0.05 were considered statistically significant.

## Results

### Treatment outcomes

**Fig. 1:**
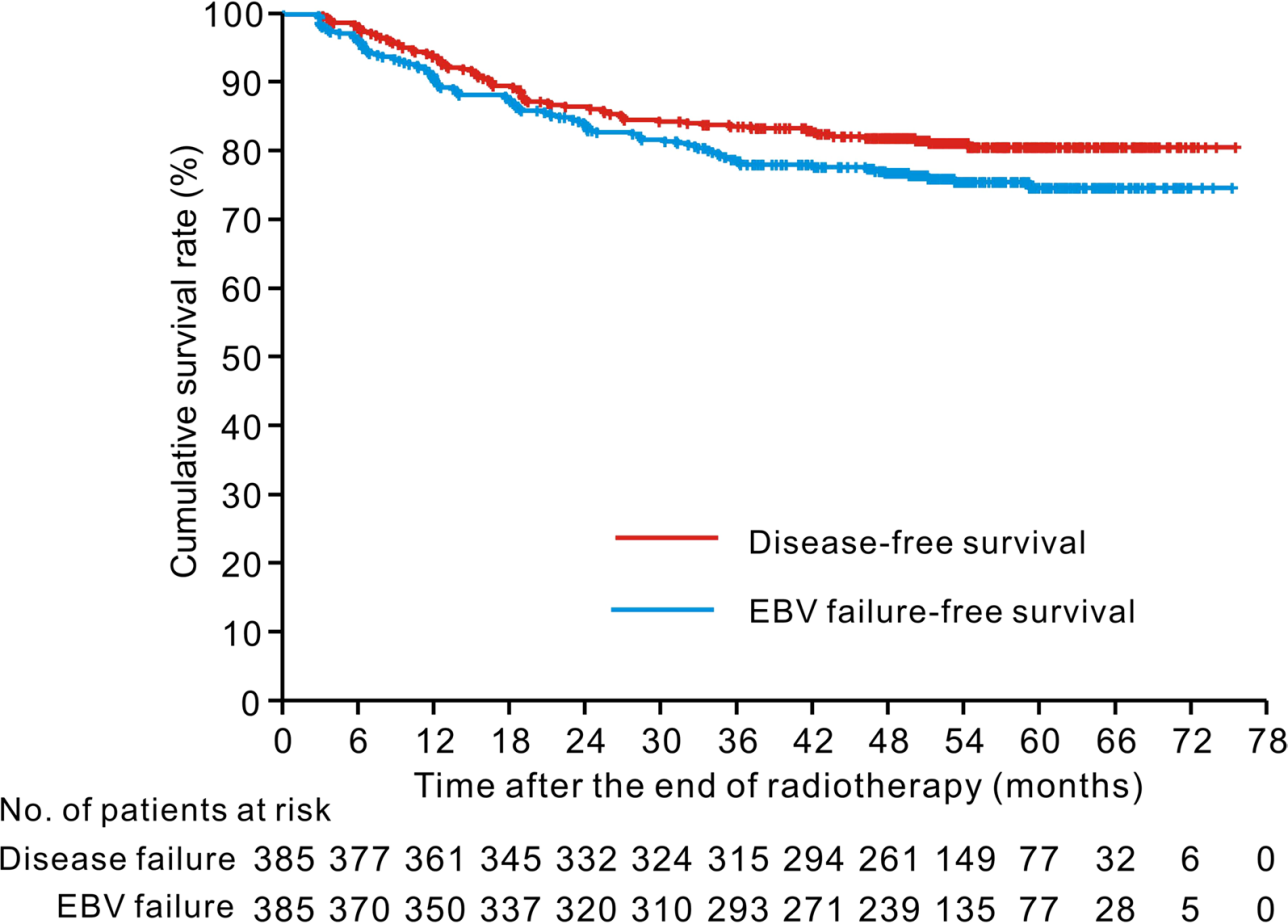
Kaplan–Meier disease-free survival and Epstein–Barr virus (EBV) failure-free survival curves for the 385 patients with nasopharyngeal carcinoma who were treated with intensity-modulated radiotherapy

A total of 385 patients were included in this study, and the clinical characteristics of these patients are shown in Table 1. The median follow-up time was 52.8 months (range 9.3–73.8 months). Seventy-two (18.7%) patients experienced tumor recurrence. The first recurrence was locoregional recurrence alone in 39 (10.1%) patients and distant metastasis with or without locoregional recurrence in 33 (8.6%) patients. The 5-year DFS, OS, LRRFS, and DMFS rates were 80.6%, 89.7%, 86.8%, and 89.3%, respectively. Plasma EBV DNA was detected in 267 (69.4%) patients before treatment and 93 (24.2%) patients during posttreatment follow-up. The 5-year EBV failure-free survival rate was 74.4%. The Kaplan–Meier survival curves for DFS and EBV failure-free survival are shown in Fig. 1.

**Table 1:** Clinical characteristics of the 385 patients with nasopharyngeal carcinoma (NPC) who were treated with intensity-modulated radiotherapy (IMRT)

Characteristic	No. of patients (%)
Sex	
Man	281 (73.0)
Woman	104 (27.0)
Age (years)	
≤ 45	235 (61.0)
> 45	150 (39.0)
Histological type	
WHO type I	3 (0.8)
WHO type II/III	382 (99.2)
T category^a^	
T1	73 (19.0)
T2	75 (19.5)
T3	168 (43.6)
T4	69 (17.9)
N category^a^	
N0	53 (13.8)
N1	244 (63.4)
N2	62 (16.1)
N3	26 (6.7)
Clinical stage^a^	
I	23 (6.0)
II	98 (25.4)
III	172 (44.7)
IVB	92 (23.9)
Chemotherapy	
No	55 (14.3)
Yes	330 (85.7)

*WHO* World Health Organization

^a^Staged according to the 7th edition of the Union for International Cancer Control and American Joint Committee on Cancer (UICC/AJCC) staging system

### Association between plasma EBV DNA status and tumor recurrence

Detectable EBV DNA during posttreatment follow-up was found in 14.4% (17/118) of patients with undetectable pretreatment EBV DNA and 28.5% (76/267) of patients with detectable pretreatment EBV DNA (*P* = 0.003). The plasma EBV DNA status during posttreatment follow-up was significantly associated with tumor recurrence in both patient groups stratified by the pretreatment EBV DNA status (detectable or undetectable) (Table 2). In all, EBV DNA was detectable during posttreatment follow-up in 12.8% (40/313) of patients who remained disease-free and 73.6% (53/72) of patients who experienced tumor recurrence (*P* < 0.001). The posttreatment follow-up plasma EBV DNA status was also associated with the site of first recurrence. Posttreatment follow-up plasma EBV DNA could be detected in 93.9% (31/33) of patients who developed distant metastasis with or without locoregional recurrence, but only in 56.4% (22/39) of patients with locoregional recurrence alone (*P* < 0.001).

**Table 2:** Association between the status of plasma Epstein–Barr virus (EBV) DNA during posttreatment follow-up and tumor recurrence in the 385 patients with NPC who were treated with IMRT and stratified by the status of pretreatment plasma EBV DNA

Site of first recurrence	Undetectable pretreatment plasma EBV DNA [*n* (%)]			Detectable pretreatment plasma EBV DNA [*n* (%)]		
	Undetectable plasma EBV DNA during posttreatment follow-up (*n* = 101)	Detectable plasma EBV DNA during posttreatment follow-up (*n* = 17)	*P*	Undetectable plasma EBV DNA during posttreatment follow-up (*n* = 191)	Detectable plasma EBV DNA during posttreatment follow-up (*n* = 76)	*P*
Any recurrence	4 (4.0)	6 (35.3)	0.001^a^	15 (7.9)	47 (61.8)	< 0.001^b^
Locoregional	4 (4.0)	4 (23.5)	0.015^a^	13 (6.8)	18 (23.7)	< 0.001^b^
Distant with or without locoregional	0 (0)	2 (11.8)	0.020^a^	2 (1.0)	29 (38.2)	< 0.001^b^

^a^ *P* values were calculated using Fisher’s exact test when any number was < 5

^b^ *P* values were calculated using the χ² test

### Failure patterns in patients with undetectable EBV DNA during posttreatment follow-up

Of the 292 patients with undetectable EBV DNA during posttreatment follow-up, 19 (6.5%) experienced tumor recurrence; the first recurrence included locoregional recurrence alone in 17 patients and distant metastasis in 2 patients (Table 2). All 17 cases of locoregional recurrence were histologically confirmed, with the exception of one case that was diagnosed by MRI and PET/CT. Two patients developed lung metastasis: one was confirmed by surgical pathology, and the other was diagnosed by chest CT and PET/CT.

### Failure patterns in patients with detectable EBV DNA during posttreatment follow-up

Of the 93 patients with detectable EBV DNA during posttreatment follow-up, 53 (57.0%) experienced tumor recurrence; the first recurrence were locoregional recurrence alone in 22 patients and distant metastasis with or without locoregional recurrence in 31 patients (Table 2). Of the 53 patients with tumor recurrence and detectable EBV DNA during posttreatment follow-up, 25 (47.2%) were histologically confirmed, whereas 28 (52.8%) were diagnosed by combined radiologic imaging methods. The other 40 patients with detectable posttreatment follow-up EBV DNA remained disease-free after a median follow-up of 29.2 months (range 0.7–65.9 months); none of these patients received preventative chemotherapy or other therapeutic interventions during posttreatment follow-up period.

Of the 53 patients with tumor recurrence and detectable posttreatment follow-up EBV DNA, 29 (54.7%) had elevated posttreatment follow-up EBV DNA levels prior to clinical recurrence, with a median interval between the observation of elevated EBV DNA level and first recurrence of 9.1 months (range 2.2–35.7 months). The median plasma EBV DNA level was 3940 copies/mL (range 40–2,420,000 copies/mL) at the time of first detection after radiotherapy, and 16,500 copies/mL (range 180–8,010,000 copies/mL) at the time of recurrence. For the 40 patient who were disease-free, the median EBV DNA level was 805 copies/mL (range 30–8840 copies/mL). Transiently detectable EBV DNA followed by a rapid regression to undetectable levels was observed in 9.4% (5/53) of patients with tumor recurrence and 97.5% (39/40) of those who were disease-free (*P* < 0.001, Table 3).

**Table 3:** Plasma EBV DNA levels in the 93 patients with detectable EBV DNA during posttreatment follow-up

Emergence of plasma EBV DNA during posttreatment follow-up	Disease free	Tumor recurrence	*P* ^d^
No. of patients	40	53	NA
Before clinical detection^a^ [*n* (%)]	NA	29 (54.7)	NA
Transiently detectable^b^ [*n* (%)]	39 (97.5)	5 (9.4)	< 0.001
Plasma EBV DNA level^c^ [copies/mL; median (range)]	805 (30–8840)	3940 (40–2,420,000)	NA
EBV DNA^c^ > 500 copies/mL [*n* (%)]	24 (60.0)	42 (79.2)	0.043
EBV DNA^c^ > 1000 copies/mL [*n* (%)]	16 (40.0)	37 (69.8)	0.004

*NA* not applicable

^a^ Emergence of plasma EBV DNA during posttreatment follow-up before clinical detection of recurrence

^b^Plasma EBV DNA was transiently detected followed by a rapid regression to be undetectable

^c^The emergence level of EBV DNA during posttreatment follow-up period

^d^ *P* values were calculated using the χ² test

### Optimal plasma EBV DNA cut-off level for predicting tumor recurrence

The plasma EBV DNA level at the time of first detection during posttreatment follow-up was analyzed as a dichotomous variable using different cut-off values (0, 500, and 1000 copies/mL). The AUC, sensitivity, specificity, accuracy, positive predictive value, and negative predictive value for each cut-off value of EBV DNA level for prediction of tumor recurrence are shown in Table 4. Compared with other cut-off values, an EBV DNA value of > 0 copy/mL had the highest AUC value (AUC, 0.804; 95% CI 0.741–0.868), with corresponding sensitivity, specificity, and accuracy of 73.6%, 87.2%, and 84.7%, respectively.

**Table 4:** Diagnostic value of plasma EBV DNA for predicting tumor recurrence in the 385 patients with NPC who were treated with IMRT

Sites of first recurrence	Emergence level of plasma EBV DNA during posttreatment follow-up	AUC (95% CI)	Sensitivity (%)	Specificity (%)	Accuracy (%)	PPV (%)	NPV (%)
Any recurrence							
	EBV DNA > 0 copy/mL	0.804 (0.741–0.868)	73.6	87.2	84.7	57.0	93.5
	EBV DNA > 500 copies/mL	0.781 (0.712–0.851)	63.9	92.3	87.0	65.7	91.7
	EBV DNA > 1000 copies/mL	0.759 (0.686–0.832)	56.9	94.9	87.8	71.9	90.5
Distant metastasis							
	EBV DNA > 0 copy/mL	0.882 (0.828–0.935)	93.9	82.4	83.4	33.3	99.3
	EBV DNA > 500 copies/mL	0.831 (0.748–0.915)	78.8	87.5	86.8	37.1	97.8
	EBV DNA > 1000 copies/mL	0.817 (0.725–0.908)	72.7	90.6	89.1	42.1	97.3
Locoregional recurrence alone							
	EBV DNA > 0 copy/mL	0.679 (0.584–0.775)	56.4	79.5	77.1	23.7	94.2
	EBV DNA > 500 copies/mL	0.684 (0.585–0.783)	51.3	85.5	82.0	28.6	93.9
	EBV DNA > 1000 copies/mL	0.660 (0.559–0.762)	43.6	88.4	83.9	29.8	93.3

*ROC* receiver operating characteristic, *AUC* area under the ROC curve, *PPV* positive predictive value, *NPV* negative predictive value, *CI* confidence interval

## Discussion

The results of the present study showed that detectable plasma EBV DNA during posttreatment follow-up could be found in NPC patients with undetectable pretreatment EBV DNA, and was significantly associated with tumor recurrence, especially distant metastasis, in patients with either detectable or undetectable pretreatment EBV DNA. Compared with other cut-off values, EBV DNA > 0 copy/mL during posttreatment follow-up had the strongest association with tumor recurrence.

Elevated levels of EBV DNA during posttreatment follow-up have been shown to associate with tumor recurrence, and play an important role in detecting and monitoring recurrence in NPC [4, 9, 14, 23–30]. However, the role of posttreatment follow-up EBV DNA assay on NPC patients with undetectable pretreatment EBV DNA has not been reported yet. In the present study, detectable EBV DNA during posttreatment follow-up was found in 14.4% of patients with undetectable pretreatment EBV DNA and was significantly associated with tumor recurrence in these patients. Our results suggested that the posttreatment follow-up plasma EBV DNA assay is also valuable in patients with undetectable pretreatment EBV DNA.

Our results showed that plasma EBV DNA could be detected in 12.8% of patients who remained disease-free during posttreatment follow-up and in 73.6% of patients who experienced tumor recurrence. The EBV DNA levels during follow-up were usually low (≤ 1000 copies/mL) and transiently detected in patients who remained disease-free, but high (> 1000 copies/mL) and detected on consecutive occasions in patients who had tumor recurrence. In the study by Hsu et al. [29], approximately 30% of patients with disease free had transiently elevated EBV DNA levels of < 400 copies/mL or no < 400 copies/mL of EBV DNA load with fluctuation. The reasons explaining transiently detectable EBV DNA remain unknown, and none of the patients with detectable EBV DNA during posttreatment follow-up in the present study had preventative chemotherapy or other therapeutic interventions before clinical diagnosis of tumor recurrence; other EBV-related diseases [29] and false-positive EBV DNA levels [32] could be the possible factors.

We found that, in patients with undetectable plasma EBV DNA during posttreatment follow-up, 6.5% of patients experienced tumor recurrence, with locoregional recurrence as the major failure pattern. Moreover, plasma EBV DNA could be detected in 93.9% of patients who had distant metastasis with or without locoregional failure, but only in 56.4% of patients who suffered locoregional recurrence as the first recurrence event (*P* < 0.001). These results indicate that plasma EBV DNA assay has a significantly lower sensitivity for detection of locoregional recurrence than for that of distant metastasis. Similar findings have been reported in previous studies: plasma EBV DNA was detected in 86%–96% of patients who experienced distant metastasis and 51%–67% of patients who suffered locoregional recurrence [4, 9, 14, 23–26, 29, 30].

One possible explanation for this phenomenon is that plasma EBV DNA levels reflects the tumor load in NPC [5, 7], and patients with locoregional recurrence usually have a lower tumor burden than patients with distant metastasis. A second explanation is that locoregionally recurrent tumor cells often regrow from irradiated tumor sites, and post-irradiation changes such as stromal fibrosis and decreased vascularity may interfere with the efflux of EBV DNA into the plasma [24]. Distant metastatic tumor cells are derived from micro-metastasis from the pre-irradiated primary tumor; thus, the release of EBV DNA from these sites is not affected by post-irradiation changes [24]. Thus, plasma EBV DNA assay is not superior to imaging modalities such as MRI scans for detecting locoregional recurrence, but may be advantageous for the early detection of metastatic disease in comparison with imaging modalities that cannot detect micro-metastasis.

The optimal threshold of posttreatment follow-up EBV DNA level for detecting tumor recurrence in NPC patients remains unclear. Hsu et al. [29] found that with the cut-off value of 400 copies/mL of EBV DNA, the sensitivity was 46%, and the specificity was 94%. Cao et al. [25] recommended 0 copy/mL as the optimal cut-off value for detecting recurrence of NPC. In the present study, we found that higher EBV DNA cut-off values had higher specificity but lower sensitivity for detecting recurrence, and the EBV DNA value of more than 0 copy/mL yielded the highest AUC in predicting tumor recurrence, with sensitivity, specificity, and accuracy of 73.6%, 87.2%, and 84.7%, respectively. The sensitivity of EBV DNA assay for predicting NPC recurrence in the present study was lower than the 74.7%–81.5% reported in other studies which also used 0 copy/mL as the cut-off value [26, 29, 30]. The possible explanation is that plasma EBV DNA assay has a high sensitivity for the prediction of distant metastasis, but not locoregional recurrence. Thus, the sensitivity for predicting tumor recurrence depends on the ratio of distant metastasis to all recurrences. In the present study, distant metastasis accounted for a relatively small portion of total recurrences (45.8%, 33/72), which lowered the sensitivity of EBV DNA in predicting tumor recurrence.

The present study has several limitations. First, the records of plasma EBV DNA levels during posttreatment follow-up were retrospectively collected, and the intervals of 3–12 months were relatively long and irregular, which could confound the results. Second, not all of the patients with tumor recurrence underwent histologic examinations or PET/CT scans. Third, selection bias may have been introduced as only patients who underwent plasma EBV DNA assays every 3–12 months during posttreatment follow-up period were recruited to this study. However, consecutive enrollment was mandated to minimize bias.

## Conclusions

Plasma EBV DNA level during posttreatment follow-up is a good marker for predicting distant metastasis, but not locoregional recurrence, in patients with NPC irrespective of the pretreatment EBV DNA status. Thus, we suggest that plasma EBV DNA assay plus nasopharyngeal and neck MRI could be an effective follow-up strategy for patients with NPC, and whole body examinations (e.g., PET/CT) should be recommended for patients with detectable EBV DNA during posttreatment follow-up. Close follow-up is needed for patients with elevated levels of EBV DNA during posttreatment follow-up but negative findings on imaging studies. The conclusions of this study require further validation in large-scale prospective studies.

## Abbreviations

NPC: nasopharyngeal carcinoma
EBV: Epstein–Barr virus
PET: positron emission tomography
IMRT: intensity-modulated radiotherapy
UICC: Union for International Cancer Control
AJCC: American Joint Committee on Cancer
MRI: magnetic resonance imaging
PFS: progression-free survival
OS: overall survival
DMFS: distant metastasis-free survival
LRRFS: locoregional recurrence-free survival
AUC: area under curve
